# Supplementary figures and images for: Modeling Brain Dynamics in Brain Tumor Patients Using the Virtual Brain
Source: eNeuro. 2018 Jun 4;5(3):ENEURO.0083-18.2018. doi: 10.1523/ENEURO.0083-18.2018 (PMC6001263; doi:10.1523/ENEURO.0083-18.2018)

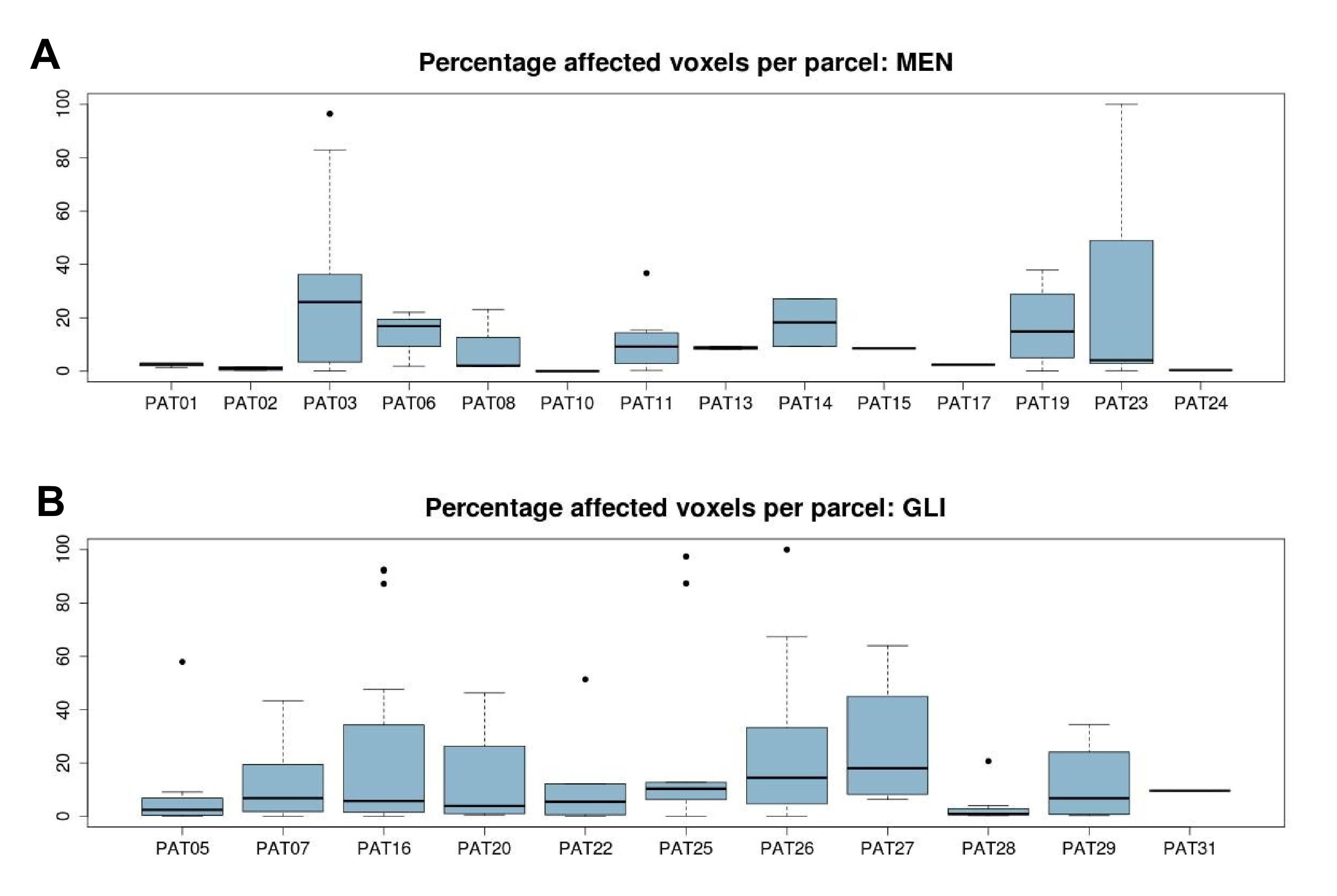

Supplement: Table 1-1 — Percentage of voxels per node that was affected by a tumor (only for tumor nodes), per subject, for meningioma patients (A) and glioma patients (B). Download Table 1-1, TIF file. [file sup_enu-eN-NWR-0083-18-s02.tif]
